# Supplementary material for: Molecular Drivers of Multiple and Elevated Resistance to Insecticides in a Population of the Malaria Vector Anopheles gambiae in Agriculture Hotspot of West Cameroon
Source: Genes (Basel). 2022 Jul 6;13(7):1206. doi: 10.3390/genes13071206 (PMC9316901; doi:10.3390/genes13071206)
Supplement: Supplementary file 1 [file genes-13-01206-s001.zip › genes-1724989-supplementary.pdf]

Supplementary material

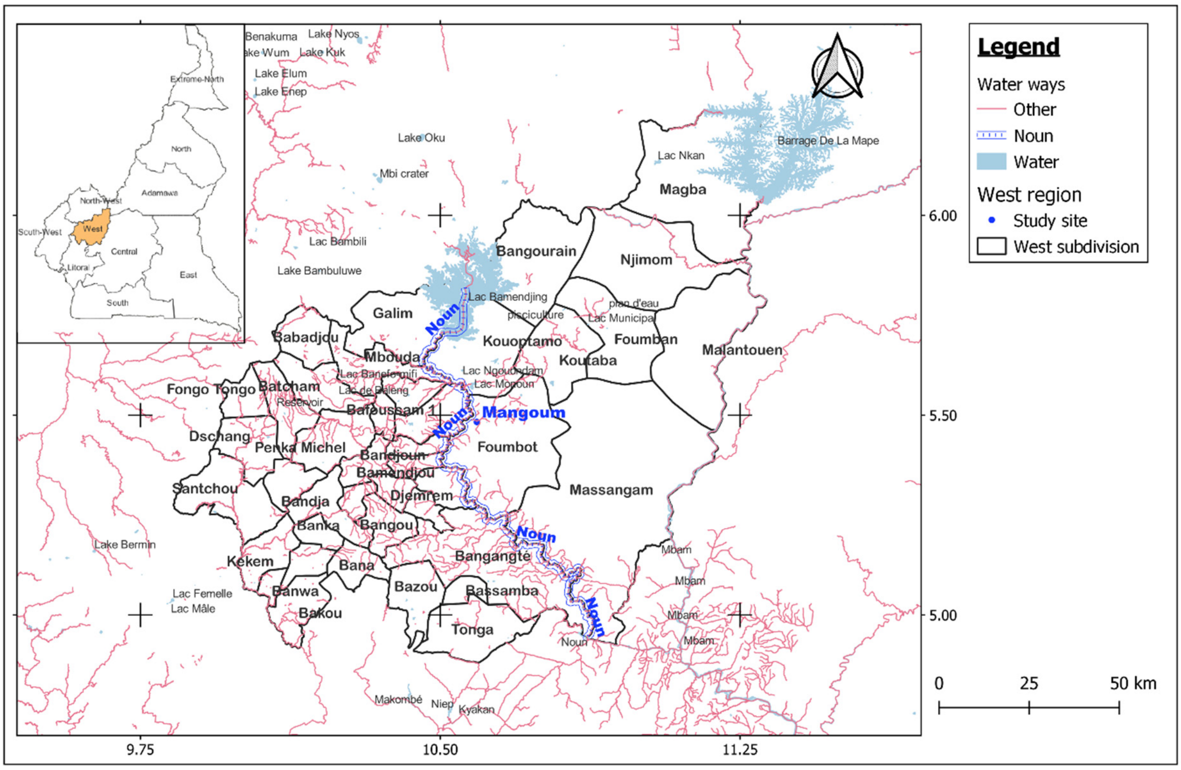

Figure S1: Map of the sampling sites.

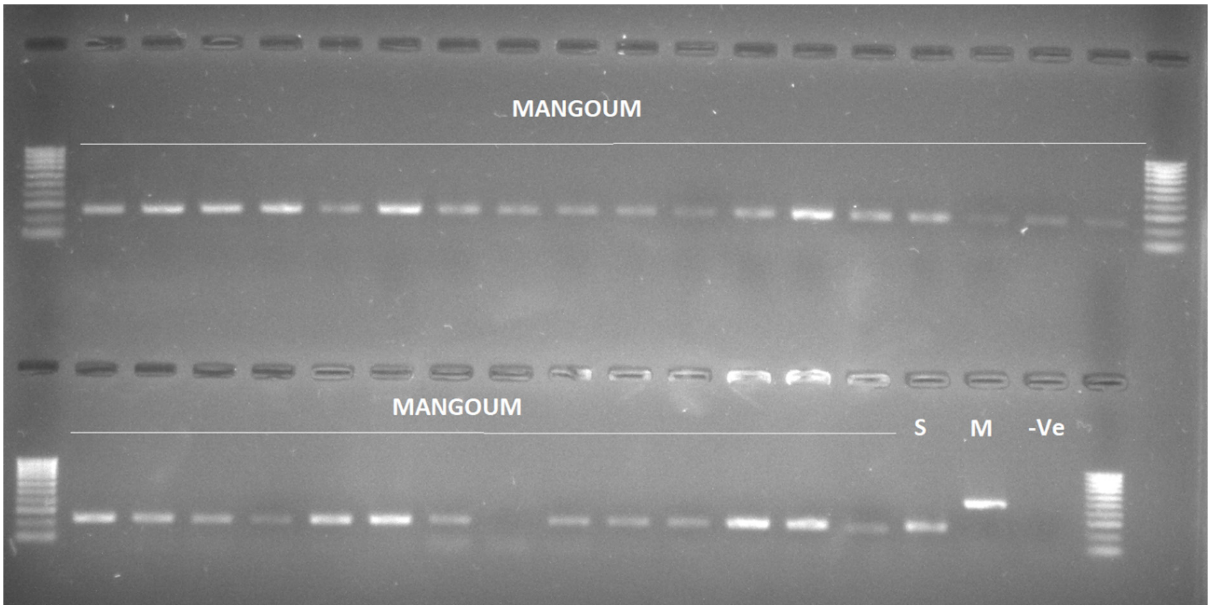

Figure S2: Electrophoresis gel of SINE PCR for identification.

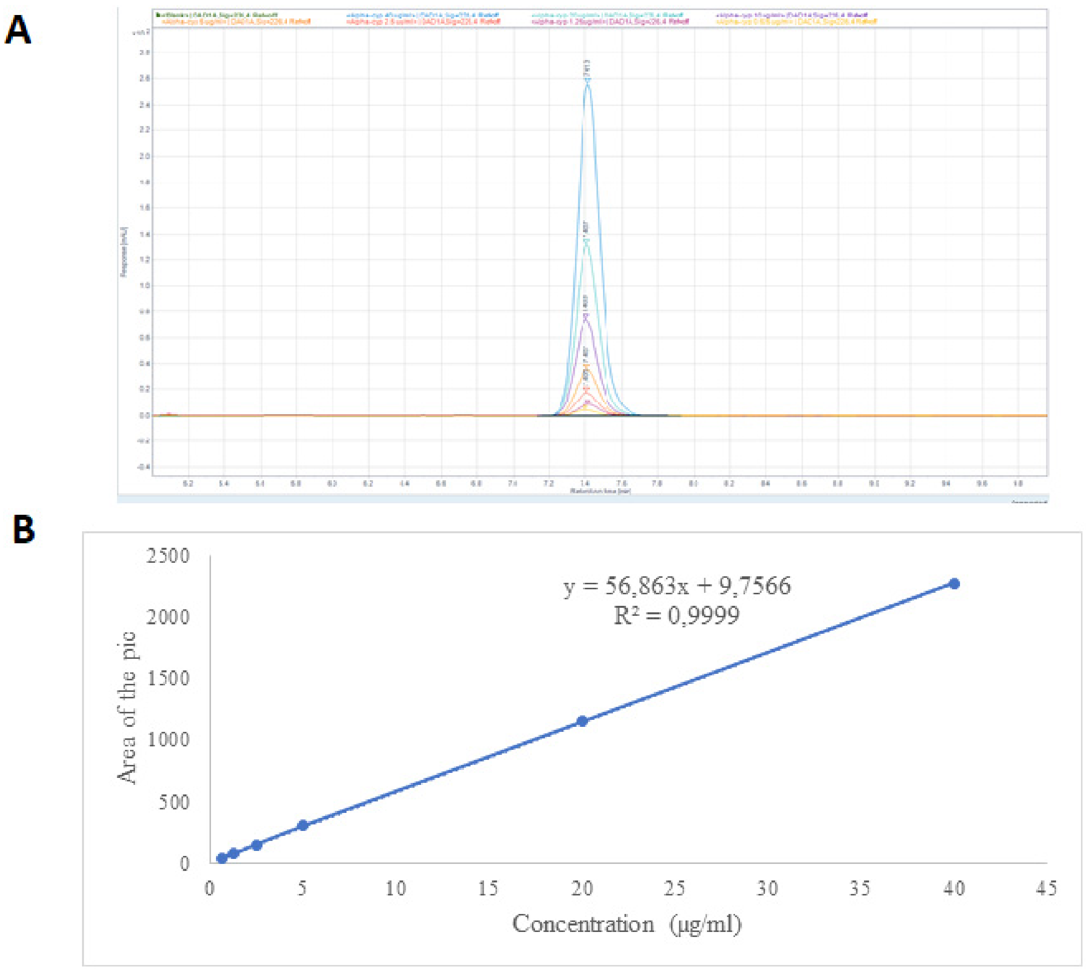

Figure S3: The chromatogram overlay of the serial dilutions of alpha-cypermethrin (**A**) and the Standard curve of alpha-cypermethrin used for concentration extrapolation (**B**).

Table S1: List of primers used

Primer set	Primer sequence (5′–3′)	Size of PCR product using cDNA template	Purpose
EFq_F	GGCAAGAGGCATAACGATCAATGCG	110 bp	Real time PCR
EFq_R	GTCCATCTGCGACGCTCCGG		
RPS7q_F	CCACCATCGAACACAAAGTTGA	117 bp	Real time PCR
RPS7q_R	TGCTGCAAACTTCGGCTATTC		
6M2q_F	CTGGCGTTGAATCCAGAGGT	144 bp	Real time PCR
6M2q_R	GATACTTGCGCAGTGATTCATTAAG		
6P3q_F	ACAATGTGATTGACGAAACCCT	101 bp	Real time PCR
6P3q_R	GGATCACATGCTTTGTGCCG		
6P1q_F	ACAGGTGGTGAACGAAACCC	75 bp	Real time PCR
6P1q_R	GGTGTAATCCTGTCCCGCAA		
9K1q_F	CCGACACGTGGTGATGGATAC	175 bp	Real time PCR
9K1q_R	CGTCGTCGGTCCAGTCAAC		
6P4q_F	CTGGACAACGTTATCAATGAAACC	84 bp	Real time PCR

6P4q_R	GCACGGTGTAATCACGCATC		
6Z1q_F	CCCGCAACTGTATCGGTCTG	138 bp	Real time PCR
6Z1q_R	TTCGGTGCCAGTGTGATTGA		
6Z2q_F	AGGCCACGAAGAACTACGAT	154 bp	Real time PCR
6Z2q_R	ACTTTTGCAGGAGTTGTGGC		
Gste2q_F	CCGGAATTTGTGAAGCTAAACC	147 bp	Real time PCR
Gste2q_R	GCTTGACGGGGTCTTTCGG		
4G16q_F	GTCCAAGAAGTTGCGTCGGAC	186 bp	Real time PCR
4G16q_R	TCTTCGATTTGCGTTGACGTG		
4G17q_F	TGTCACGACTACATGAGCGA	157 bp	Real time PCR
4G17q_R	CGCAGGTGGATCTTCAGTTG		
SAP1_F	TTTGATCCGGAGAACAAGTAC	92 bp	Real time PCR
SAP1_R	CGTTCTTCCGGGTTTCCA		
SAP2_F	CTACTTCAAGTGCCTGATGG	250 bp	Real time PCR
SAP2_R	CTTGATGCCCTCCTTCTTG		
SAP3_F	GAGAAGCAGAAGAGTGGCAC	150 bp	Real time PCR
SAP3_F	ACAGGTTGATGCCCTTCTTC		
Plas_F	GCTTAGTTACGATTAATAGGAGTAGCTTG		TaqMan
Plas_R	GAAAATCTAAGAATTTCACCTCTGACA		
Falcip+FAM	TCTGAATACGAATGTC		
OVM+HEX	CTGAATACAAATGCC		
